# Air Pollution and Its Potential Consequences for Tourism and Career Development from Students’ Perspective: A Case Study of the Gdańsk Agglomeration in Poland

**DOI:** 10.3390/ijerph20032651

**Published:** 2023-02-01

**Authors:** Magdalena Bogalecka, Aleksandra Grobelna

**Affiliations:** 1 Department of Industrial Products Quality and Chemistry, Gdynia Maritime University, 81-87 Morska Str., 81-225 Gdynia, Poland; 2 Department of Marketing and Quantitative Methods, Gdynia Maritime University, 81-87 Morska Str., 81-225 Gdynia, Poland

**Keywords:** air pollution, Gdańsk agglomeration, tourism and hospitality students, tourist attractiveness, career development

## Abstract

The purpose of this study is to present the state and variability of air pollution and its potential consequences for the intensity of tourism traffic based on the example of the Gdańsk agglomeration as a very popular tourist destination of Northern Poland. Specifically, the study seeks to answer the question how a future, qualified tourism workforce, such as tourism and hospitality (T&H) students from higher educational institutions (HEIs) located in the investigated area, perceive the problem of air pollution and whether their perception may have a potential impact on their attitudes and career aspirations towards working in the T&H industry after graduation. In this study, both a desk-research method and a questionnaire were used. The main results reveal that although the intensified tourist traffic does not coincide with high concentrations of pollutants and a poor quality of air, it cannot be clearly stated that tourists choose a destination being guided by the condition of ambient air pollution. The findings also show that T&H students are strongly aware of the air pollution problems and its negative consequences for the perceived attractiveness of a tourist destination and its labour market. To the best of the authors’ knowledge, this study is among the first to analyse the relationships between air pollution and students’ perceptions of its consequences for tourism and for career development in the tourism industry, which is highly dependent on the environmental quality.

## 1. Introduction

Many countries all over the world make a real effort to develop the tourism industry [1], perceiving it as an impulse to dynamize the socio-economic development of the country [2]. Tourism in Poland, as an element of the service sector, plays a significant role in generating new jobs and creating an income [3]. Moreover, the economic importance of tourism is also manifested in improving the quality of life of local communities and in increasing the competitiveness of a region [2]. At the same time, tourism contributes to discovering the most valuable cultural and environmental resources which improve the internal and external image of the country, regions or cities [2].

The features of the current civilization such as permanent stress, dramatically fast speed of life, and mental and physical exhaustion create a deep need for rest and taking advantage of contact with nature and climatic values [4]. All these aspects may contribute to increasing numbers of tourist arrivals for leisure purposes [5] where the tourist experience may be enhanced by clean air at the destination [6]. Likewise, exposure to air pollution in the visited destination may lead to serious health problems and could be a serious threat, decreasing the quality of that experience. Depending on their concentration, pollutants may cause mild effects, impaired lung function, respiratory symptoms, the necessity to take medication, reduction of physical activity, hospitalization and finally death [7,8]. As a result, tourist arrivals to such destinations may decrease, as many potential tourists may cancel their tourism plans to such places.

However, despite the indisputable fact that the environment determines the development of tourism [9] and affects the quality of the tourist product [5,10,11,12], there is still insufficient research concerning the influence of the environment on tourism, specifically the impact of air quality [13]. Meanwhile, the problem of air pollution and its consequences for tourism development is particularly vital [14], especially compared with residents of polluted destinations, tourists are much more sensitive to acute effects of this state [15]. As a result, health risk as an important component of travel risk that may have a negative impact on potential tourists’ intentions to visit a particular destination [16], making it no longer attractive. This has been confirmed with results of many previous studies, which proved that the state of air pollution may affect the level of tourist attractiveness in view of the potential tourists (e.g., [17,18,19]) and which may influence the economy of a given city with consequences to its labour market.

Undoubtedly, tourism is an important generator of jobs [20]. Some even emphasize that generating employment is usually the most direct and beneficial impact of tourism on the host community [21]. However, with a decrease in the perceived attractiveness of a certain destination, its labour market is also likely to experience some related difficulties, as the ensuing drop in tourist traffic may result in a reduction in tourism jobs that are no longer needed in a particular region. Consequently, the tourism industry in such places is losing its appeal as an employer and ceases to be perceived as an attractive place for future career development. This seems critical given the chronic problem of high employee turnover in the tourism industry generally [22,23,24], and in Poland specifically [22], and the fact that many young people, particularly T&H students, are not interested in T&H jobs, and many of them do not want to enter the industry upon graduation [25,26], perceiving such jobs as *short-lived professions* [27]. Hence, seeking answers to the question whether air pollution may potentially threaten future career development in the T&H industry seems to be particularly vital [28], as on the one hand, more and more cities are struggling to maintain official air quality standards [17], which may seriously affect its tourist attractiveness and, consequently, its labour market, and on the other hand, tourism labour is still an area of relatively scanty research despite an obvious need to be able to manage and plan for tourism labour requirements [20]. Therefore, identifying obstacles for career development in T&H, particularly from the perspective of T&H students as the future workforce trained in the field, is still a fundamental but understudied topic [25]. Combining the above areas of interest is rarely, if at all, a focus of scientific research, as many previous studies mainly relate to the problem of air pollution in the context of tourist attractiveness through tourists’ assessments, whereas studies regarding the perception of other tourism stakeholders, such as future T&H workforce, seem to be limited, if any. Thus, this study fills the existing theoretical gap by offering a new perspective on understanding the problem of air pollution and its negative consequences for tourism and career development from the viewpoint of T&H students.

Based on the above, the aim of the study is to present the state and variability of air pollution [29,30] and its potential consequences for the intensity of tourism traffic based on the example of the Gdańsk agglomeration as a very popular tourist destination that with the neighbouring Gdynia and Sopot agglomerations, is also called the Tri-City agglomeration (Poland). Additionally, the purpose of this study is to investigate whether and how the perception of air pollution may have a potential impact on attitudes and employment aspirations of a future, qualified tourism workforce, such as T&H students from higher educational institutions (HEIs) located in the investigated area.

Because tourism triggers significant income for many destinations [17], creating a pool of jobs directly and indirectly supported by the industry, these study results might be of vital importance for both tourist entities, local authorities and educational institutions.

## 2. The Gdańsk Agglomeration as a Tourist Destination of Northern Poland

Polish voivodships are highly diversified in terms of tourism resources, infrastructural facilities and accessibility [3]. However, the literature underlines that the most intense tourist traffic is still developing in northern Poland, including the Pomeranian voivodship [15], which belongs to the group of regions with the greatest tourist potential [3] and which is one of the most often chosen destinations for holidays, placing it at the forefront of the country [31].

It is underlined that the Pomeranian region is characterized, in particular, by the excellent natural values and landscapes. There are many legally protected areas and objects which make up the system of nature protection [2]. The Pomeranian voivodship is also well known for its very attractive Tri-City agglomeration consisting of Gdańsk, Sopot and Gdynia, which are among the most visited tourist centres in Central Europe, where among many attractions, everyone will find something for themselves, including a mix of sports, cultural and entertainment events [32].

Gdańsk is the oldest of the three cities and has a very rich history [33]. With its over 1000 years of tradition, Gdańsk is one of the most recognizable Polish cities that takes a special place in the minds of tourists whose increasing number has been observed over the years [34]. It is a very attractive tourist centre with a strategic geographic location—at the Baltic Sea coast, in the vicinity of the charming Kashubian Lake District, Kociewie, Hel Peninsula and Malbork. Additionally, numerous congress, fair and exhibition events, especially those dedicated to one of the greatest local assets of the city, amber, make Gdańsk one of the most attractive tourist destinations within the south Baltic Sea Region [34].

The strong brand of Gdańsk in the international arena contributes to the recognition of the entire Pomeranian region and its cities making up the metropolis [31]. Thus, Gdańsk was among the top three recommended places to visit in the “European Best Destination 2017” competition, named by TripAdvisor as one of the TOP Rising Destinations 2018 [31].

The tourist attractiveness of Gdańsk is also confirmed by the number of tourists visiting this city. In the summer of 2021 (June–August), there were 1,105,662 tourist arrivals. For comparison, in 2019, the number was 1,098,430, while in the heavily pandemic year of 2020, it was 960,000 [35]. Domestic tourists accounted for slightly over 80% of the tourist traffic, whereas the remaining ones were foreign tourists, mainly Germans—22% and Scandinavians—14% [35]. Visiting guests highly ranked the attractiveness of their stay in the city in 2021—it was at the level of 8.8 on a 10-point scale [35].

Analysing the main purposes of the visits, tourists mostly declared for leisure—54%, then visiting friends and families—15.1% and business issues—10.2% [35]. They spent their free time focusing mainly on sightseeing, walking and resting on the beach [35]. Hence, due to spending much time outside, air quality may become an important factor conditioning the tourists’ perception of urban space and even affect their decisions regarding the choice of a particular destination [17].

## 3. Air Pollution as a Threat to Tourism Development

Human health is the greatest wealth and the most valuable asset that determines people’s safety [36]. It is often underlined that people can lose jobs, homes or even all of their money, but if they lose their health, then they lose everything. Tourism development is largely dependent on many factors, including the natural environment [37]. Undeniably and undoubtedly, the quality of the environment has a great significance for tourist traffic [15], particularly today, when many people live under heavy stress, and one of the main motivations for traveling is to seek a place with a pleasant environment to recover and relax [16]. Therefore, environmental quality seems to be an important factor in potential tourists’ decision-making processes [16]. However, today’s tourist arrivals may create some difficulties in the context of tourists’ well-being and health, particularly in cities, as in many regions, the level of air pollution is relatively high enough [15] to create a serious threat to health safety [36,38] by causing respiratory and circulatory system diseases, and even cancer [36].

The term *air pollution* is defined as the presence of harmful and toxic solid, liquid or gaseous substances as well as their mixtures in a given layer of the atmosphere in concentrations that are burdensome for humans or/and have a negative impact on their health and quality of life [36]. On the other hand, the World Health Organization (WHO) defines *air pollution* as contamination of the indoor or outdoor environment by any chemical, physical or biological agent that modifies the natural characteristics of the atmosphere [39]. The sources of air pollution are divided into natural, resulting from processes taking place in nature, and anthropogenic, which are related to organized human activity. These include emissions from mobile sources related to the transport of motor vehicles and fuels, emissions related to home heating in the municipal and housing sector, etc. [36]. The levels of concentrations of pollutants in the air directly result from the volume of emissions into the atmosphere and the current meteorological conditions in a given area [36].

According to the WHO, in many of the world’s largest cities, air pollution levels are significantly increasing [40,41]. The WHO data show that 99% of the world-wide population breathes air that exceeds the recommended limits [41]. The Organization for Economic Co-operation and Development (OECD) reports that by 2050, air pollution, just in cities, will have become the leading environmental cause of mortality in the world [34]. This is similar in the European Union, where air pollution constitutes the greatest threat to people’s health [13]. Unfortunately, Poland belongs to the group of European countries with the highest levels of air pollution [42]. The level of air purity has significantly deteriorated, covering greater areas of the country and generating a serious growth in health hazards [36]. In many Polish cities, the air pollution norms and the permissible standards are often exceeded [36]. As a result, both citizens and visitors are exposed to air pollution [15].

Based on the above, it is not surprising that, among many other risks, traveling includes those connected with the possible negative health outcomes of air pollution [40]. Travellers arriving in cities, especially during an air pollution episode, are particularly exposed to its large concentration; moreover, they may lack the necessary adaptation, precautionary measures or advice on how to minimize the associated health risks [38,40]. Such relatively sudden exposure to air pollution could have an adverse effect on the travellers’ cardiopulmonary system [40]. Although limited, the results of previous studies illustrate an association between exposure to elevated levels of air pollution and its negative health outcomes for travellers [38]. Air pollution can cause many diseases and health problems that do not always require long exposure to harmful factors [17]. For example, it was proven that travelling to polluted cities, even for short-term exposure, negatively affects health, as is particularly visible in respiratory symptoms or cardiovascular events [38,40].

Understandably, tourists can also feel the negative effects of breathing polluted air, and because tourist trips are rarely mandatory (except for business trips), people who are aware of this may consider the condition of air quality as one of the factors determining attractiveness of a particular destination influencing their final decision to choose it as a place of potential visit [17]. Therefore, air pollution emerges as a vital problem in the tourism industry [13,15,18,19,43,44,45,46,47,48,49,50,51,52,53,54,55,56], decreasing the quality of tourist experience, which is fatal to the development of tourism destinations in the long run [16]. It is underlined that countries and cities with poor air quality may lose their attractiveness, and consequently, tourists who fear for their own health may start to avoid them [15]. Thus, it is important for destinations to maintain high environmental quality to attract visitors and to increase their tourism competitiveness [16].

## 4. Assessment of Air Pollution

Air pollution and its health risks are commonly evaluated based on the concentration of some representative pollutants, such as sulphur dioxide—SO_2_, carbon monoxide—CO, nitrogen dioxide—NO_2_, ozone—O_3_, benzene—C_6_H_6_, and particulate matter with an aerodynamic diameter less than 2.5 µm or between 2.5 and 10 µm—PM_2.5_ and PM_10_, respectively [39]. However, these indicators are used to assess different emission sources from various sectors and kinds of pollution. Namely, CO is a more suitable indicator of road traffic, whereas PM_2.5_ and PM_10_ are considered to be the more appropriate indicators of resuspension of road dust [57]. In other words, it means that one pollutant can reach a danger level because of its high concentration, while simultaneously, another one can be at a harmless level in the same place.

Sulphur dioxide is a colourless gas, non-inflammable and denser than air with a stifling and pungent odour detectable at 0.5 ppm, although concentrations above 6 ppm have been reported to produce instantaneous mucous membrane irritation. It is easily soluble in water; therefore, it combines with water vapor in the atmosphere to produce acid rain that has a negative impact on human and animal health, as well as on plant life, and causes destruction (corrosion) of materials. SO_2_ can affect human health, particularly in those who suffer from chronic lung diseases and asthma. It can also irritate the eyes. The main anthropogenic sources of SO_2_ emission are from sulphur-containing fossil fuel when it is being burned.

Ozone is a colourless or bluish unstable gas with a characteristic odour. In the environment, O_3_ contributes to *smog* when its ground-level layer is formed primarily from photochemical reactions between other pollutants: nitrogen oxidizes and volatile organic compounds, e.g., benzene and presence of sunlight. Therefore, the highest concentration of O_3_ is observed during a sunny and hot summer season. O_3_ is a highly reactive oxidizer that is dangerous to health and destroys materials. When inhaled, O_3_ causes an inflammatory response in the eyes and the respiratory tract, leading to pulmonary injury.

Benzene is a colourless liquid with a gasoline-like odour. It belongs to volatile organic compounds that are emitted with vehicle exhaust gases. C_6_H_6_ causes chronic health effects such as cancer, central nervous system disorders, kidney and liver damage, reproductive disorders, as well as birth defects.

Carbon monoxide is a colourless, odourless and tasteless toxic gas that is released into the atmosphere as a result of incomplete combustion of fuels, coal and other organic compounds with limited oxygen presence. CO is harmful when inhaled in large amounts, as it combines with haemoglobin and blocks oxygen, making it ineffective to deliver oxygen to bodily tissues.

Nitrogen dioxide is a reddish-brown gas with a characteristic odour that is toxic when absorbed by the skin or inhaled. NO_2_ is a secondary product formed from nitrogen and oxygen present in the atmosphere during high-temperature combustion of coal, fuel, oils, etc. (the primary product is nitrogen oxide—NO). Therefore, road traffic is the principal source of nitrogen oxides, collectively known as NO_x_. Their concentrations are the highest in urban areas where traffic is the heaviest. NO_2_ combines with water vapor in the atmosphere to produce *acid rain*.

Particulate matters can be made up of many different chemicals and contain microscopic liquid droplets or solids that are small enough to be inhaled and cause health problems. The main source of airborne PM_2.5_ and PM_10_ in cities is road traffic emissions, especially from diesel vehicles. The impact of PM depends on its size and the number of particles retained in various areas of the respiratory system. PM_2.5_ is smaller and more likely to travel and penetrate the deepest sections of the lungs, where it can accumulate or dissolve in biological liquids, causing asthma aggravation, impaired lung activity, and acute respiratory responses. PM_10_ is more likely to deposit on the surfaces of the larger airways of the upper region of the lung.

The limit values of the abovementioned pollutants constitute the air quality indicators and the background of the air quality assessment (Table 1).

## 5. Material and Methods

The research consists of two main parts. The first one concerns the concentration of air pollutants in the Gdańsk agglomeration, and their analysis corresponds to the standards in force in Poland, as presented in Section 3. The second part presents the research conducted among students of the HEIs offering T&H courses and located in the Gdańsk agglomeration. The aim of this part of the research is to empirically determine T&H students’ attitudes, as the future industry-qualified workforce, towards air pollution and its consequences for:

- the perceived attractiveness of tourist destinations;
- students’ career aspirations for employment in the T&H industry;
- tourists’ attitudes and behaviours in the visited places (this part of the research refers only to students declaring working experience in the industry and having direct contact with tourists).

**Figure 1:**
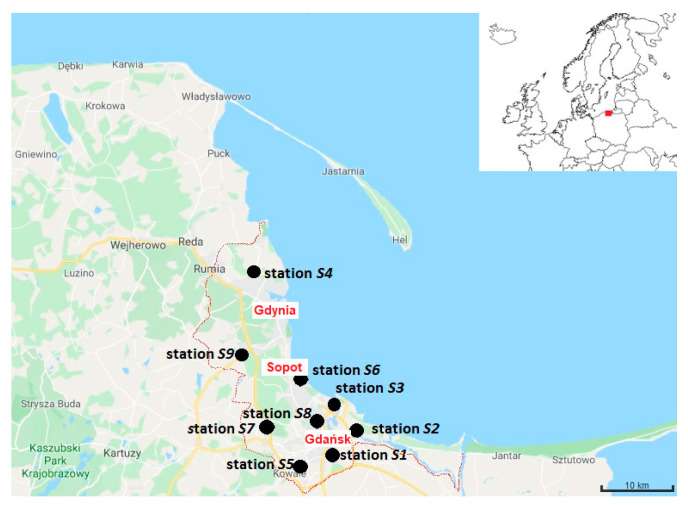
Location of air pollution monitoring stations within the Tri-City agglomeration (the agglomeration consists of Gdańsk, Gdynia and Sopot, bounded with the red dotted line). Source: Own elaboration.

Referring to the first part of the research, it is crucial to underline that the Tri-City agglomeration has over 1 million inhabitants and is affected by pollution from the shipbuilding industry and transport, as well as from domestic sources. The regional and automatic air monitoring network continuously measures (commonly counted every hour) the concentration of all or selected pollutants mentioned above within the Tri-City agglomeration and collects data from 9 monitoring stations. Five of them are located in Gdańsk, three in Gdynia, and one in Sopot (Figure 1). An additional monitoring station is located in Tczew, outside the Tri-City agglomeration. The monitoring network is controlled and accepted by the Regional Inspector of Environment Protection and the State Sanitary Officer, and data are freely accessible through the website of the Chief Inspectorate for Environmental Protection, Poland [58].

In this research, the data on the concentration of particular air pollutants come from the monitoring stations located in a distance of approximately 10 km from each other (Table 2). The selection of the stations was guided by access, if possible, to continuous data in the analysed period (some stations were temporarily out of service) and close proximity to each other.

Next, the estimated volume of tourist traffic in Gdańsk divided into individual months was calculated on the basis of research on the tourist traffic of the Gdańsk Tourist Organization and the Prof. B. Synak Pomeranian Scientific Institute in Gdańsk and based on the average occupancy of accommodation establishments, based on data collected by the Gdańsk Tourist Organization [60].

Both the results of the concentration of particular air pollutants and the intensity of the tourist traffic come from the same 7-year period (2015–2021). This enabled their comparison and determined whether tourists come to the agglomeration when the air pollution is high and whether they take the pollution status into account when choosing a tourist destination.

The second part of the research has involved T&H students. The respective HEIs were selected based on their offer of tourism and/or hospitality courses, on their location in Gdańsk and on the cooperation/familiarity between researchers and these HEIs.

Specifically, the location of Gdańsk is important for a number of reasons. Firstly, Gdańsk and Tri-City are a well-known and very attractive tourist destination on the coast of the Baltic Sea in Northern Poland [25], where tourism development is particularly dependent on leisure values, including good quality of air. Thus, unsurprisingly, the Gdańsk agglomeration also has a well-developed and complex air monitoring system that allows for controlling the intensity of air pollution and provides regular measurement information. Secondly, Gdańsk is considered as a major academic and business centre in Pomerania [61,62] that educates future employees for the needs and requirements of the tourism industry. On the other hand, it dynamically creates new jobs in the industry, particularly in the summer season [61,62].

Data have been collected using an online questionnaire created in the Google Forms application, which is an easy-to-use survey administration tool distributed to students via the Microsoft Teams platform. The latter is a cloud-based web service containing a set of tools and services for team collaboration, supporting the educational process in the HEIs participating in this study. The respondents were requested to open the link sent to them and to fill in the questionnaires during their classes, as agreed upon with the teachers of the participating HEIs. The students were assured that their participation in this study was voluntary and anonymous and that their answers would be confidential. They were also reassured that their participation was not a formal part of their study program [63]. A pilot test had been conducted before to verify good understanding of the survey instrument, and its appropriateness was confirmed by feedback. Ultimately, 162 usable surveys were obtained. The Statistical Package for the Social Sciences and Microsoft Excel applications were used for statistical analysis.

## 6. Results

The recorded concentrations of air pollutants come from the monitoring stations presented in Table 2, from the 7-year period, i.e., 2015–2021. Only the concentrations of CO were always classified as *very good* during the experimental time. The concentrations of other pollutants were generally classified as *very good* (C_6_H_6_—98.66%, SO_2_—98.05%, NO_2_—92.29%, O_3_—80.75%, PM_10_—68.31%, PM_2.5_—59.20% of the total time) or *good* (PM_2.5_—35.59%, PM_10_—26.28%, O_3_—18.89%, NO_2_—7.67%, C_6_H_6_—1.26%, SO_2_—1.21% of the total time), but there were some records for these pollutants classified as *moderate* (PM_2.5_—4.14%, PM_10_—3.96%, SO_2_—0.58%, O_3_—0.36%, C_6_H_6_—0.07%, NO_2_—0.05% of the total time) or *sufficient* (PM_10_—1.04%, PM_2.5_—0.85%, SO_2_—0.08%, C_6_H_6_—0.01% of the total time) during the research period. Only the concentrations of PM_10_, PM_2.5_ and SO_2_ exceeded the *bad* and *very bad* pollution levels. There were a few incidents of no more than 7 h in September 2018 and February 2019 for SO_2_, and some cases of no more than a few days, mainly in the fall, the winter and the spring seasons of the considered period (see Figures 5–11).

**Figure 2:**
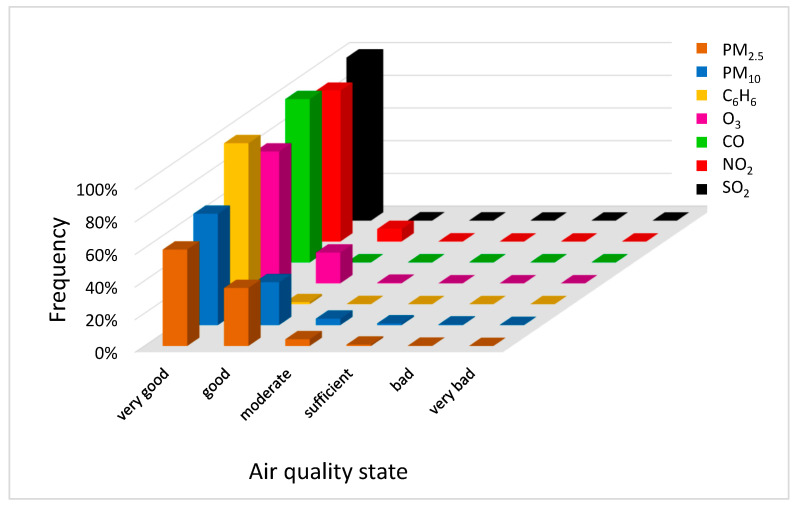
Frequency of particular air pollutants at air quality states (the Gdańsk agglomeration, years 2015–2021). Source: Own study based on data of the Chief Inspectorate for Environmental Protection [58].

The above results show that CO is not a good indicator of air pollution, as it does not quantify the level of pollution in contrast to other pollutants, especially PM_10_ and PM_2.5_ that are characterized with the most variation (Figure 2).

**Figure 3:**
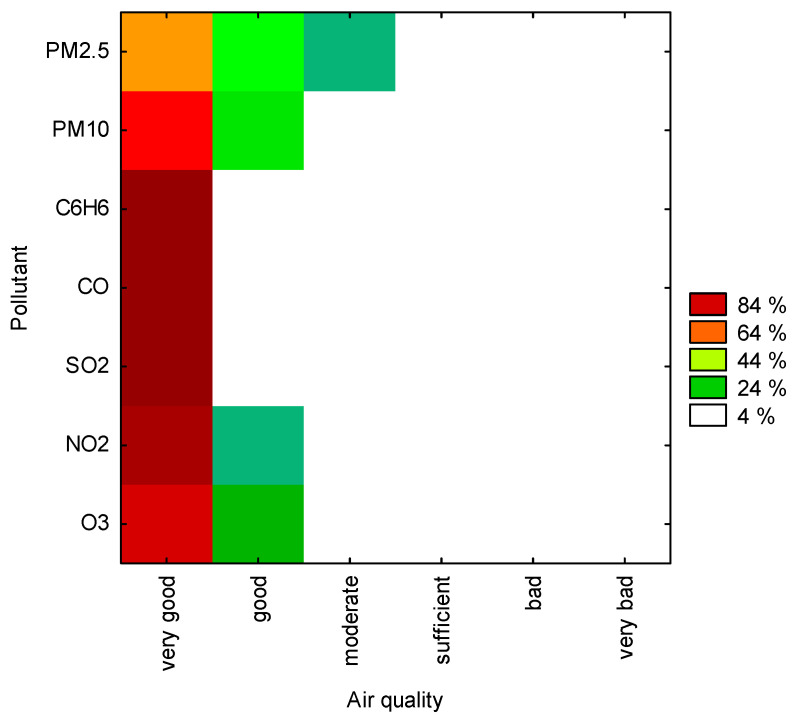
Similarities between air quality and chemical compound/particulate matter concentrations in percentage, using two-way joining cluster analysis. Source: own study.

A similar statement can be drawn on the basis of a two-way joining cluster analysis conducted to identify similarities between air quality (as variables) and pollutant concentrations expressed in percentage (as cases). The two-way joining cluster analysis was performed using Statistica 13.3 software (TIBCO Software Inc., Palo Alto, CA, USA), and the results are presented in Figure 3. This is a contour chart where the colours from green, through orange, red, to brown indicate increasing clusters. The legend to the right of the chart points at a given level of air quality. The results of the two-way joining cluster analysis show that air quality was most often *very good* or *good* (left side of the chart). The best air quality was in the case of C_6_H_6_, CO and SO_2_, as well as NO_2_, and the concentrations of these pollutants were similar to each other (dark brown fields).

The mentioned analysis is applied when both variables and cases can be expected to form clusters simultaneously. A difficulty in interpreting the results of this analysis may arise from the fact that similarities between different clusters may lead to the formation of different subsets of variables. For this reason, the structure of clusters is by nature not homogeneous; however, this method can be considered a powerful data exploration tool [64].

**Figure 4:**
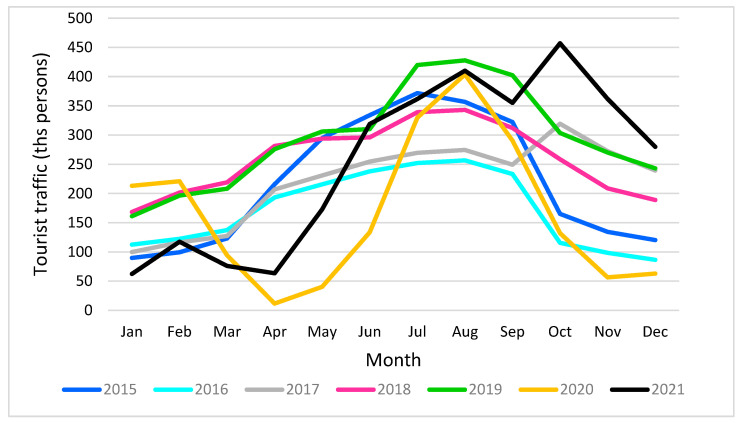
Intensity of tourist traffic changes (2015–2021) in the Gdańsk agglomeration. Source: own study.

An analysis of the intensity of the tourist traffic shows the highest peaks for the summer months of June–August while the traffic begins to increase from the end of March/early April and decrease at the end of September/early October (Figure 4). As the literature underlines, these temporary changes in the volume of tourist traffic may depend on natural conditions, such as the level of temperature and rainfall, the day length, etc. [12,65], and local resources of tourist values and tourist infrastructure [10]. In the same vein, by the sea, in a temperate climate, such as in Gdańsk, the tourist season lasts about 2–3 months in the summer [10], which is also confirmed by the statistical data showing that many tourists come to Tri-City, usually to Gdańsk, in the summer season, mainly for leisure purposes. This may confirm attractiveness of the costal location of the city and its natural climatic conditions. The nature of demand, but also natural values of a given destination, results in the seasonal use of tourism supply [66]. For example, the average occupancy of tourism accommodation establishments, specifically hotels in Gdańsk, according to the Gdańsk Tourist Organization’s statistics, during the year ranges from about 54% in the winter months (December–February) to about 87% during the summer months (June–August) [60,67], which confirms that natural factors, especially climatic ones, could be the strongest determinants of these changes with which the rhythms of tourists’ arrivals coincide [68,69].

The data presented in Figure 4 also reflect that the tourism sector has been mostly affected because of COVID-19. According to the World Tourism Organization (UNWTO), in the first quarter of 2020, when the lockdown in most countries started, the intensity of international tourist arrivals declined by 22% on the global scale and by 19% in Europe [70]. Figure 4 with the narrowest yellow peak in comparison to the other ones proves that tourism was an especially affected sector in 2020, because of closed hotels, grounded planes and travel restriction on the global scale [71,72,73,74,75,76,77]. However, compared to previous years 2015–2018, a relatively high peak in 2020 results from almost only one possibility in traveling, which was domestic tourism, due to restrictions and difficulties in international travel.

**Figure 5:**
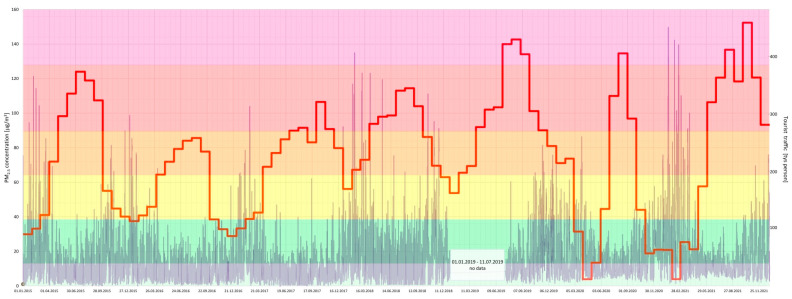
PM_2.5_ concentration in relation to tourist traffic (the Gdańsk agglomeration, years 2015–2021). Source: own study.

**Figure 6:**
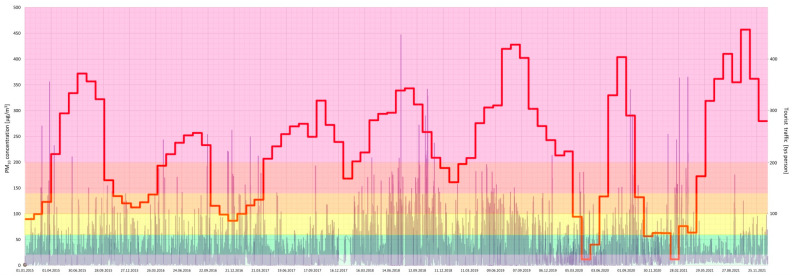
PM_10_ concentration in relation to tourist traffic (the Gdańsk agglomeration, years 2015–2021). Source: own study.

**Figure 7:**
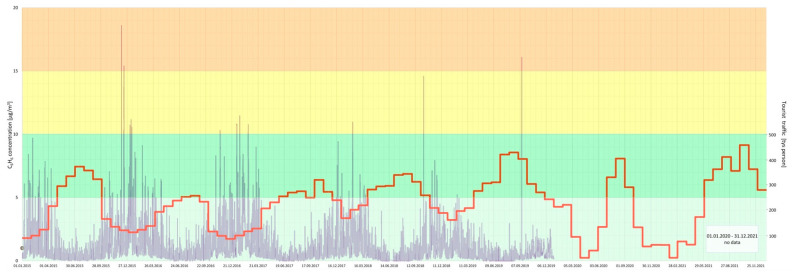
C_6_H_6_ concentration in relation to tourist traffic (the Gdańsk agglomeration, years 2015–2021). Source: own study.

**Figure 8:**
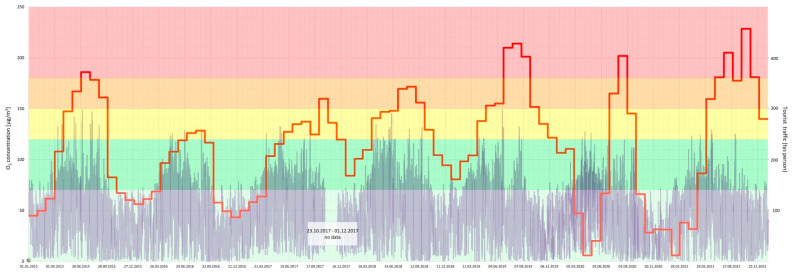
O_3_ concentration in relation to tourist traffic (the Gdańsk agglomeration, years 2015–2021). Source: own study.

**Figure 9:**
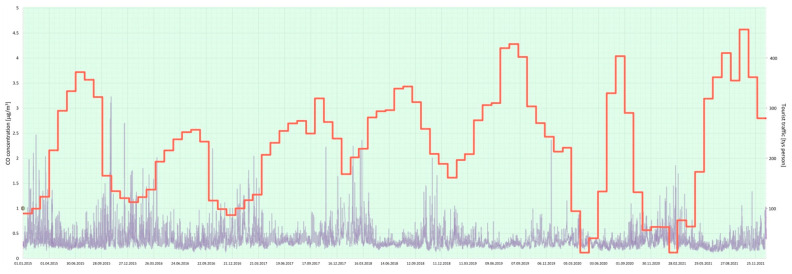
CO concentration in relation to tourist traffic (the Gdańsk agglomeration, years 2015–2021). Source: own study.

**Figure 10:**
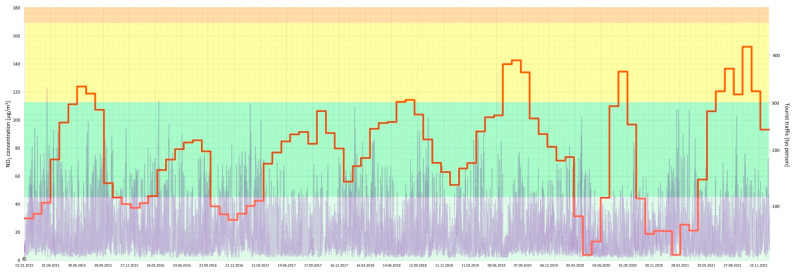
NO_2_ concentration in relation to tourist traffic (the Gdańsk agglomeration, years 2015–2021). Source: own study.

**Figure 11:**
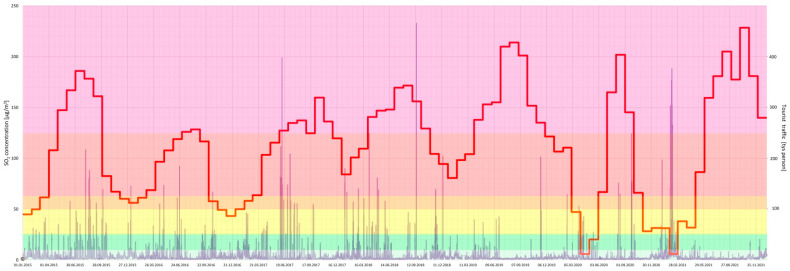
SO_2_ concentration in relation to tourist traffic (the Gdańsk agglomeration, years 2015–2021). Source: own study.

Furthermore, a joint analysis of the concentration of particular air pollutants and the intensity of tourist traffic changes over a 7-year period (2015–2021) in the Gdańsk agglomeration was performed (Figure 5, Figure 6, Figure 7, Figure 8, Figure 9, Figure 10 and Figure 11).

The results of this detailed analysis are presented in Figure 5, Figure 6, Figure 7, Figure 8, Figure 9, Figure 10 and Figure 11, where the changes in concentrations of particular air pollutants are characterized by many variables and are marked with a grey line, whereas the intensity of the tourist traffic is marked with a red one. The colours of fields in Figure 5, Figure 6, Figure 7, Figure 8, Figure 9, Figure 10 and Figure 11 correspond to those of the pollution levels and air quality given in Table 1.

It follows from the above analysis that generally more intense tourist traffic is observed during the period when pollutant concentrations (except O_3_) were at lower (safe) levels. However, the analysis shows that PM concentrations mostly approached or reached dangerous values (*bad* or *very bad*) during the considered time (Figure 5 and Figure 6). Fortunately, the *bad* and *very bad* air qualities according to particulate matters are usually observed during the winter, when the intensity of tourist traffic decreases after the holidays surge. Only 2018 shows the highest number of incidents of *bad* and *very bad* PM_10_ status during the high season, when tourist traffic was at its highest (Figure 6).

The high concentration of ground-level O_3_ results from more sunlight and higher air temperatures typical in the summer. The high concentrations of O_3_ in parallel with an increase in tourist traffic, i.e., in the summer, may be of concern (Figure 8). On the other hand, higher concentrations of O_3_ are recorded on sunnier and warmer days; therefore, its presence anywhere in the summer is rather unavoidable.

The CO level was always classified as *very good*, and the NO_2_ level was *very good* or *good* during the discussed time (Figure 9 and Figure 10). Thus, these pollutants are not seen as a problem in the Gdańsk agglomeration. Nevertheless, higher values of their concentrations (but still *very good* or *good*) are observed in the winter, probably because of the heating season. Additionally, changes in CO and NO_2_ concentration are observed each day, as their concentration also depends on the intensity of road transport, which is heavy during the day and disappears in the evening and at night.

A similar trend is observed with regard to C_6_H_6_: its highest concentration (but commonly at a *very good* or *good* and incidentally *moderate* level) also probably due to the heating season and during the low tourist season is observed (Figure 7).

SO_2_ is a pollutant that, in addition to PM, also reached hazardous levels: *bad* and *very bad* (Figure 11). It might be suspected that high SO_2_ concentrations should be recorded during the winter and the heating season. However, it is surprising that this was not always the case, as a dangerous level of SO_2_ was visible in the summer months (e.g., August 2015, June 2016, June–July 2017 or September 2018).

Although the intensified tourist traffic does not coincide with high concentrations of pollutants and poor air quality, on the basis of the above analysis, it cannot be clearly stated that tourists chose a destination because they were guided by the state of ambient air pollution. However, it is reassuring that during high tourist traffic, the air quality was quite good, except for incidents that usually lasted up to a few hours, although Gdańsk, as well as many other cities, performs a number of other functions, and implementation of some of them may cause side effects, e.g., in the form of emissions of substances contributing to air pollution [17]. Furthermore, the location of the Gdańsk agglomeration near the sea is a favourable circumstance, as water has the ability to absorb and remove pollutants, such as CO, SO_2_, and NO_2_, from the air. Moreover, frequent winds in this area prevent pollutants from accumulating in one place.

Regarding the results of the second part of the research, an analysis of the respondents’ profiles shows that female respondents accounted for the majority (72.8%) of all surveyed students; 44.4% of the respondents were aged 22–25 years, whereas slightly less (43.2%) were between 18 and 21 years old. The remaining ones (12.3%) were above 25 years of age. Most of the students (70.4%) declared having working experience in the tourism industry.

In the first part of the questionnaire, students were asked about their perception of the air pollution problems and its consequences for tourist traffic and the attractiveness of a destination. As regards the question whether students have ever been interested in the air pollution problems (Table 3), the study results revealed that most of them have been interested in that issue (76.5%). Only a small percentage of the respondents confirmed lack of such interests (14%) or did not take an unequivocal position towards the analysed subject (14.8%).

Analysing students’ attitudes towards the air pollution consequences for tourism (Table 4), the overwhelming majority of the students confirmed that air pollution of a given region may have serious consequences for lowering its tourist attractiveness (86.4%) and for reducing the tourist traffic (84%) in such a region. Only a small percentage of the respondents did not see such threats for the lowering of a region’s tourist attractiveness (4.9%) and for reducing its tourist traffic (6.1%).

In the question: “Do you think that the air pollution in the given region could lead to tourists’ dissatisfaction with their stay and spreading negative opinions about the polluted place?”, most of the T&H students confirmed that it could rather (49.4%) or absolutely (38.3%) diminish tourists’ satisfaction with their vacation, also leading to negative opinions about such destinations (85.2%).

Of particular interest in this study is also investigating whether air pollution may have consequences for students’ perceptions, as the future T&H workforce, of the industry attractiveness as an employer and their future career aspirations towards T&H (Table 5). The study results revealed that more than half of the study respondents (54.3%) believed that air pollution in a given region may have an impact on the reduction of jobs in the tourism industry in that destination. Furthermore, the same percentage of students (54.3%) took the position that they would not associate their professional career in tourism with such a place. Subsequently, Spearman’s rank correlation has been computed to assess the relationship between the perceived consequences of air pollution for the labour market in tourism (reduction in number of the jobs) and the students’ perceptions of their future careers within a region of high air pollution. The results demonstrate a negative and significant correlation between these two variables (*r_s_* = −0.301, *p* < 0.001). In other words, students who believed that air pollution might have an impact on the reduction of jobs in the tourism industry statistically more often declared that they would not associate their career in tourism with a region with high air pollution. This can create a significant challenge, especially for the tourism industry, which already experiences high employee turnover rates [78,79].

**Figure 12:**
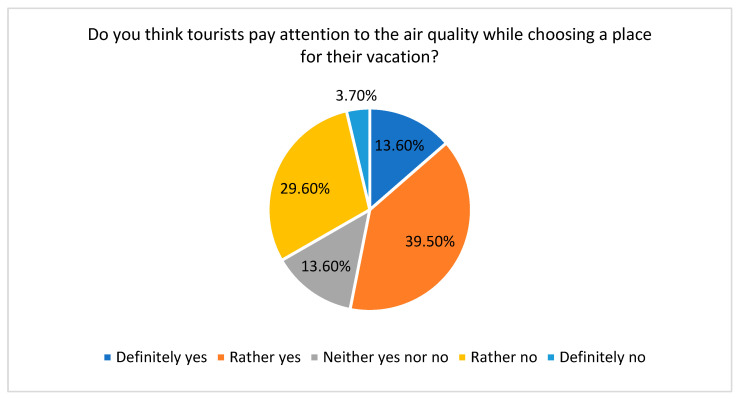
Tourists’ sensitivities to air quality while choosing a vacation destination. Source: own study.

**Figure 13:**
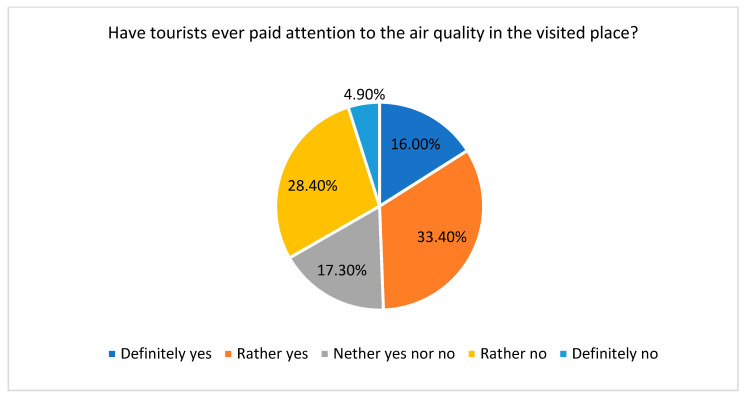
Tourists’ sensitivities to air quality in visited places. Source: own study.

The last part of the survey was devoted to learning about students’ experiences of working with tourists according to their attitudes and behaviours connected with the air quality in visited places (Figure 12 and Figure 13). Most of the students with working experience (53.1%) indicated that, in general, tourists paid attention to the air quality when choosing a vacation destination; in other words, as noted in the literature, air quality is a factor considered by tourists in making a decision about their destinations [80].

Moreover, almost half of the surveyed students (49.4%) confirmed that tourists also paid attention to the air quality during their stay at the given place, for example, through asking about the level of air pollution in a particular destination (Figure 13).

**Figure 14:**
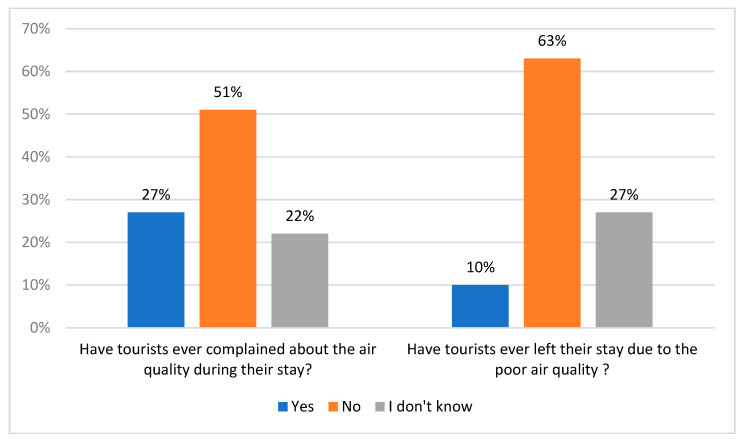
Consequences of air pollution for tourists’ stays in view of the respondents’ experiences. Source: own study.

Additionally, 27% of the students with working experience also pointed out that tourists served by them complained about the air quality during their stay, and moreover, 10% of the students experienced tourists’ resignation from their stay in a given destination because of the poor air quality (Figure 14).

Summing up, T&H students who work in T&H reported their experience with tourists’ sensitivities to air quality, which may broaden their perspective on the air pollution problem and its consequences.

## 7. Discussion

The main results of this study, given the example of Gdańsk, reveal that the intensified tourist traffic does not occur simultaneously with the poor state of the air caused by high concentrations of pollutants. On the other hand, it cannot be clearly stated that tourists choose a destination based on the state of ambient air quality and its possible pollution. However, analysing T&H students’ perception of the problem of air pollution, the findings show that young people participating in this study are strongly aware of its negative consequences for the perceived attractiveness of a tourist destination and its labour market. This view seems to cover tourists’ perception on that issue, because as proven in findings of previous studies, air pollution may affect the tourists’ assessments of the level of tourist attractiveness of a given destination and thus affect decisions concerning their tourist trips [17,18,19], which finally may have its consequences for the economic condition of a given region, resulting in a reduction in tourism-related jobs.

Respondents mostly pointed out that air pollution might lead to reducing tourist traffic, tourists’ dissatisfaction, spreading negative opinions about a particular region and decreasing the number of jobs in tourism. Most students also declared that they would not associate their career in tourism with a region with a high level of air pollution. This may result from perceived job instability in polluted regions. As proven in extensive previous research among T&H students, perceived job instability may reduce students’ working intentions in the tourism industry (e.g., [25]) decreasing its attractiveness for career development from the perspective of a future workforce [26,27,28,81], which may have huge financial and psychological implications for T&H organizations [82]. Therefore, reinforcing protection and adequate management of the tourism environment seems to be a critical issue to achieve sustainable tourism development [37] and to assure the quality of human resources that heavily depend on having educated, well-trained, skilled and committed employees [23].

Based on the above, this study answers the literature voices for more research on perceptions and attitudes of T&H students towards their future careers in the tourism industry [81,82,83,84,85], focusing on factors that may potentially prevent a future, qualified workforce from finding employment in tourism upon graduation. Presumably, this study is among the first to investigate potential relationships between air pollution and students’ views on its consequences for both tourism and their career prospects in the industry, which already experiences high employee turnover rates, and which faces shortages of trained and skilled employees [81,86,87,88].

This study also provides several practical recommendations that also could be applied in other tourist regions facing air pollution problems. Firstly, it is particularly vital to take care of regional resources, including the natural ones, such as air quality, that may help tourists to rest, regenerate and reinforce, contributing to building the competitiveness of the region and its attractiveness in the visitors’ eyes. However, one should remember that the ever-changing environmental conditions resulting from human activity require environmental knowledge and commitment from society. Only an environmentally literate society will be able to behave respectably and responsibly towards the environment [37]. Therefore, any actions in the field of education are needed, and any activities increasing social environmental responsibility and awareness are strongly recommended [89]. However, along with educational activities aiming to raise awareness of the impact of pollution on health, actions aimed at improving air quality in cities are also necessary.

Therefore, it is important to constantly monitor the air quality of the Gdańsk agglomeration and of other tourist destinations. Although a specific monitoring network exists in many cities, including Gdańsk where it functions as part of the Monitoring of the Atmosphere of the Tri-City Agglomeration project [34], the results of the air quality tests should be broadly published and targeted not only for the inhabitants but also for travellers to inform them about the level of air pollution. Such information should be easily accessible and available. Thus, it is proposed to disseminate it via different sources, including the internet, those related to tourism (e.g., official websites of tourist entities, tourist information centres, accommodation establishments, etc.) and via developing and implementing mobile applications that will inform about the level of air quality in a particular destination. Such widespread access to these data may contribute to tourists taking an interest in the state of the air and making a more conscious choice of their holiday destination. Furthermore, tourists who care about a healthy reset and who know the state of air pollution and are aware of the threats can put pressure on the local authorities to contribute to improving air quality with legal, economic, and technical tools at their disposal. On the other hand, tourist entities should put more pressure on the local authorities, which might be a causative factor in investing in tools related to air protection [5], particularly in such regions where the tourist function intertwines with the industrial one, as is the case of Gdańsk. Local authorities should also significantly educate the local community in the field of limiting emissions of pollutants, influencing pro-ecological attitudes and behaviours by increasing environmental awareness and implementing adequate administrative and technical solutions to increase the quality of air and hence the quality of life in a particular region. Specifically, to improve the air quality, it is absolutely necessary to implement the so-called *anti-smog* resolutions, including financial support for replacing heating systems to eliminate coal boilers and furnaces [90].

It is also generally recommended for tourists to avoid traveling to highly polluted cities [40]. However, if tourists decide to travel, it is important to educate them on how to prepare to visit particular destinations during air pollution episodes and how to minimize the associated health risks. For example, travellers who plan to visit cities should take several precautionary measures (such as researching pollution levels in the destination cities or avoiding seasons with high pollution levels), as they may experience symptoms or changes in the lung function that might affect their quality of life and health [38].

The results of this study also showed that tourist destinations, including Gdańsk, should take care of air quality, not only to increase their attractiveness in visitors’ eyes but also to attract and maintain a young, qualified workforce, which is a chronic problem of the tourist industry worldwide, including Poland [91].

Summarizing, actions undertaken in a given tourist destination aimed at limiting emissions will probably be able to influence its tourism development and its attractiveness not only in the eyes of the visitors, but also in the eyes of the young qualified workforce who will be likely to work in that particular region, providing excellent service for visitors.

## 8. Conclusions

The aim of this study was to present the state of air pollution and its potential consequences for the intensity of tourism traffic based on the example of the Gdańsk agglomeration as a very popular tourist destination of Northern Poland. Moreover, this study sought to answer the question of how the problem of air pollution can be perceived by future, qualified tourism workforce such as T&H students, and whether their perception may have potential impact on their attitudes and their employment aspirations towards working in the T&H industry upon graduation.

The obtained results constitute a significant contribution to the knowledge about the factors influencing students’ career perceptions in T&H [82,84,85,92], offering a new perspective on understanding the employment aspirations in tourism through the prism of very timely topics, namely the problem of air pollution and its negative consequences increasingly experienced by societies around the world and seriously threatening tourism development, which is strongly dependent on environmental quality. This paper draws attention to the fact that problems arising from air pollution can affect residents [93] and tourists visiting a particular destination [6,14,16] but may also influence its labour market, decreasing its attractiveness in the eyes of future qualified workforce.

This study also has some limitations. Due to the fact that the research was conducted only among students of selected HEI located in the investigated area, the results of this study cannot be generalized. Therefore, future studies with a larger sample of tourism and hospitality students from other HEI located in different geographical regions of Poland are recommended.

As to the directions of future research, one should bear in mind that although tourism can potentially benefit the environment by contributing to its protection and conservation via raising environmental awareness and serving as a source of financing the protection of natural areas and increasing their economic importance [94], the tourism industry also visibly affects the environment both directly and through supporting industries [5,12,95]. As is pointed out, tourism can be a source of the same types of pollution as any other industry, including emissions of air pollution, noise, litter, release of industrial and municipal sewage, chemicals, and even architectural/visual pollution [94,96,97]. There are also voices that emissions of air pollution induced by tourism are greater than from other service sectors [6]. Therefore, tourism development may damage the natural environment, and the results of previous studies revealed that there is a link between the intensity of tourist traffic and the level of air pollution with PM_10_ [15]. Hence, it could be highly interesting in future studies to investigate how (if at all) the tourism industry may influence the quality of air in the Gdańsk agglomeration or another city in a different location, and what kind of tourist traffic creates the greatest source of this kind of pollution. Nevertheless, one should remember about the dual role of tourism as a *culprit* and a *victim* of environmental degradation, which lowers the tourism attractiveness of a destination and, consequently, earnings from leisure or travel [9].

As to the limitations of this study, this research concentrates on air pollution in the Gdańsk agglomeration; thus, future studies should be conducted in other tourist destinations to compare the achieved findings, to seek similarities and differences, and for its potential sources, also in relation to other factors important from the tourist point of view (e.g., water quality of secluded bathing areas). Moreover, due to the case-study nature of the research and the fact that this study has been conducted only on a small sample of T&H students from selected HEIs, the results cannot be generalized to all T&H students in Poland (including Tri-City), and the conclusions may concern only the investigated group of students. They would become more precise with a more numerous group of respondents. Thus, future research in a broader educational setting with a larger sample from different destinations in Poland or even from different countries is recommended to obtain a more representative sample of T&H students and to determine if the students’ perceptions of the issue of air pollution and its consequences may vary along with geographical and even cultural differences. Yet, despite that limitation, the current findings may constitute a basis for discussion and a starting point that will stimulate further extended research.

## Figures and Tables

**Table 1:** Air quality according to the concentration of pollutants.

Air Quality State	Pollutant’s Concentration (μg/dm^3^)						
	SO_2_	CO	NO_2_	O_3_	C_6_H_6_	PM_2.5_	PM_10_
very good	0–50	0–2∙10^3^	0–40	0–70	0–5	0–12	0–20
good	50.1–100	2.1∙10^3^–6∙10^3^	40.1–100	70.1–120	5.1–10	12.1–36	20.1–60
moderate	100.1–200	6.1∙10^3^–1∙10^4^	100.1–150	120.1–150	10.1–15	36.1–60	60.1–100
sufficient	200.1–350	1.1∙10^4^–1.4∙10^4^	150.1–200	150.1–180	15.1–20	60.1–84	100.1–140
bad	350.1–500	1.41∙10^4^–2∙10^4^	200.1–400	180.1–240	20.1–50	84.1–120	140.1–200
very bad	>500	>2∙10^4^	>400	>240	>50	>120	>200

Source: Based on the Chief Inspectorate for Environmental Protection [58].

**Table 2:** Location of monitoring stations and the measured pollutants.

Station #	Geographic Coordinates	Measured Pollutants
2	54°22′04″ N, 18°42′04″ E	C_6_H_6_
3	54°24′03″ N, 18°39′27″ E	PM_10_
8	54°22′49″ N, 18°37′13″ E	O_3_, CO, PM_2.5_, NO_2_, SO_2_,

Source: Own elaboration based on the specifications of monitoring stations [59].

**Table 3:** Students’ interests in air pollution problems.

	Answer:	Definitely Yes	Rather Yes	Neither Yes Nor No	Rather No	Definitely No	Total
Question:							
Have you ever been interested in the air pollution problems?		36 22.2%	88 54.3%	24 14.8%	8 4.9%	6 3.7%	162 100%

Source: own study.

**Table 4:** Perceived consequences of air pollution in the respondents’ opinions.

	Answer:	Definitely Yes	Rather Yes	Neither Yes Nor No	Rather No	Definitely No	Total
The Main Consequences of Air Pollution in a Given Destination Are							
Lowering tourist attractiveness of a place		82 50.6%	58 35.8%	14 8.6%	8 4.9%	0 0%	162 100%
Reducing tourist traffic		62 38.3%	74 45.7%	16 9.9%	8 4.9%	2 1.2%	162 100%
Tourists’ dissatisfaction		62 38.3%	80 49.4%	16 9.9%	2 1.2%	2 1.2%	162 100%
Negative opinions		48 29.6%	90 55.6%	14 8.6%	10 6.2%	0 0%	162 100%

Source: own study.

**Table 5:** Consequences of air pollution for career perceptions in the T&H industry.

	Answer:	Definitely Yes	Rather Yes	Neither Yes Nor No	Rather No	Definitely No	Total
Question:							
Can air pollution in a given region reduce the number of jobs in the tourism industry in that region?		20 12.3%	68 42.0%	44 27.2%	28 17.3%	2 1.2%	162 100%
Would you associate your future career with tourism in a given region, knowing that it is a place with high air pollution?		2 1.2%	20 12.3%	52 32.1%	58 35.8%	30 18.5%	162 100%

Source: own study.

## Data Availability

The data presented in this study are available on request from the corresponding author.
